# Biochemical effect of a histidine phosphatase acid (phytase) of *Aspergillus japonicus* var. Saito on performance and bony characteristics of broiler

**DOI:** 10.1186/s40064-016-3082-8

**Published:** 2016-08-24

**Authors:** Alexandre Maller, Thays Cristina Oliveira de Quadros, Otto M. Junqueira, Alfredo Lora Graña, Ana Paula de Lima Montaldi, Ricardo Fernandes Alarcon, João Atílio Jorge, Maria de Lourdes T. M. Polizeli

**Affiliations:** 1 Departamento de Bioquímica e Imunologia, Faculdade de Medicina de Ribeirão Preto, Universidade de São Paulo, A. Bandeirantes, 3900, Ribeirão Preto, SP 14040-901 Brazil; 2 Departamento de Zootecnia, Faculdade de Ciências Agrárias e Veterinárias, Universidade Estadual Paulista Júlio de Mesquita Filho, Jaboticabal, SP 16050-680 Brazil; 3 FATEC Indústria de Nutrição e Saúde Animal LTDA, Arujá, SP 07400-000 Brazil; 4 Departamento de Biologia, Faculdade de Filosofia, Ciências e Letras de Ribeirão Preto, Universidade de São Paulo, Ribeirão Preto, SP 14040-901 Brazil

**Keywords:** Biochemical effect, Phytate, Phosphate, Animal diet, Supplementation

## Abstract

Phytases are enzymes that hydrolyze the ester linkage of phytic acid, releasing inositol and inorganic phosphate. The phytic acid (phytate) is a major form of phosphorus in plant foods. Knowing that diet for animal of production has the cereal base (corn and soybean), primarily, broilers need for an alternative to use of the phosphate present in these ingredients, since it does not naturally produce the enzyme phytase, which makes it available. The aims of this work was studding the safe supplementation of *Aspergillus japonicus* var. Saito crude phytase in feeding broilers and check the biochemical effect on performance and bones of these animals. The enzymatic extract did not have aflatoxins B1, B2, G2 and G1 and zearalenone and ochratoxin, and low concentrations of this extract did not have cytotoxic effects on cells derived from lung tissue. The in vivo experiments showed that the phytase supplied the available phosphate reduction in the broiler feed formulation, with a live weight, weight gain, feed intake, feed conversion, viability, productive efficiency index and carcass yield similar to the control test. Furthermore, the phytase supplementation favored the formation of bone structure and performance of the broilers. The results show the high biotechnological potential of *A. japonicus* phytase on broiler food supplementation to reduce phosphorus addition in the food formulation. So, this enzyme could be used as a commercial alternative to animal diet supplementation.

## Background

Phytases are able to release phosphates from phytic acid. This is one of the major forms of phosphorus (P) in animal feeds of plant origin, representing 60–90 % of total P content in plants. Phytases hydrolyze phytate into one molecule of inositol and six molecules of inorganic phosphate in a sequential dephosphorylation reaction (Greiner et al. 2007; Yao et al. 2012). So, the addition of phytase in broiler feed could increase the bioavailability of phosphorus for this monogastric animal.

The greatest biotechnological advances in animal feeding are the discoveries of novel ingredients and supplements associated with the adjustments of nutritional requirements. In this way, the enhancement of formulation of animal feed and efficiency optimization of low-quality raw materials will certainly produce benefits for animal feeding. The formulation of animal diet consists of a mixture of several components, such as soybean, corn, wheat bran and others. These components are rich in phosphorus in the form of phytate, and the supplementation of this enzyme may increase the available phosphorus. The enzymatic additives have an indirect function on animal diet. They assist the digestive process, increasing the bioavailability of the nutrients present in food (Kebreab et al. 2012). So, such additives can be incorporated into animal feed, aiming at enhancing their performance and, thus, profitability.

Fungal enzymes have several advantages to industrial applications, such as thermal and pH stabilities. The phytase of *A. japonicus* is thermostable up to 50 °C, with a t_50_ of 40 min, and it has more than 80 % of activity at 41–42 °C and acid pH (Fonseca-Maldonado et al. 2014). These conditions are found in digestive tract of broiler and indicate a good performance of this enzyme in supplementation of animal feed. In this way, previous works showed positive effects in vitro of the supplementation of this enzyme on broiler food (Promdonkoy et al. 2009; Maller et al. 2014), but no works were found in the literature about effects in vivo.

Then, the aims of this work were to study the safe supplementation of crude phytase from *A. japonicus* in broiler feed and verify the biochemical effect on performance and bones of these animals.

## Methods

### Organism and growth conditions in submerged fermentation (SbmF)

The stock cultures of the fungal strain *A. japonicus* Saito (Facchini et al. 2011) were kept in slants of PDA solid medium and maintained at 4 °C. Further, the fungus was incubated on PDA solid medium at 30 °C for 7 days before use in cultivation and enzyme production. The SbmF was carried out by the inoculum of 4 × 10^6^ conidia into 2-L Erlenmeyer flasks containing 500 mL of liquid medium, with 10 g/L wheat bran. The cultures were incubated at 30 °C under orbital stirring (100 rpm) for 72 h. Then, the cultures were filtrated through a Whatman No. 1 filter paper in a Buchner funnel. The filtrate was saved as a source of crude phytase.

### Enzymatic assays and protein determination

Phytase activity was determined using ammonium molybdate method (Yin et al. 2007) modified by Maller et al. (2014), using as substrate 10 mg/mL phytic acid dodecasodium (Sigma-Aldrich, St. Louis, MO). A standard curve was generated using 100–1000 µM KH_2_PO_4_ solutions. One phytase unit (FTU) was defined as the amount of enzyme that released 1 µmol of inorganic phosphorus from phytic acid dodecasodium per minute under the assay conditions. The crude phytase extract used in the assays also contain 38, 7.8 and 6.8 U/mL of β-xylosidase, α-glucosidase and CMCase, respectively. Protein was assayed according Lowry et al. (1951), using bovine serum albumin as standard. The specific activity was defined as FTU/mg of protein.

### Cytotoxicity assay and mycotoxins

The cellular proliferation assay was performed using 25, 50 and 100 % of *A. japonicus* crude phytase and MRC-5 cell line derived from normal lung tissue, according Montaldi and Sakamoto-Hojo (2012). Each experiment was performed in duplicate wells and repeated three times. The doxorubicin (0.3 µg/mL) treatment was used as positive control of toxicity and saline 0.9 % was used as negative control. The analysis of aflatoxins B1, B2, G1 and G2 and total zearalenone and ocratoxin A was performed by JLA Brasil Laboratory (Marília, SP, Brazil) from crude phytase of *A. japonicus* growth in Czapek medium, with 1 % of wheat bran (w/v) at 30 °C under agitation (100 rpm) for 72 h.

### Characteristics of broilers, plants and handling

The assays were carried out with a total of 1050 1-day-old Cobb male broiler. The broilers were accommodated in experimental masonry shed, with tile roof and concrete floor. The sidewalls had 3.20 m in height with wire mesh, and external mobile curtain. The shed was divided into 80 boxes of 3.10 × 1.05 m, which were separated by screens 0.70 m in height. In the first 2 weeks of bird ages, infant tubular feeders and aluminum waterers were used, which were gradually replaced by a tubular feeder with a capacity of 20 kg of feed and bell drinkers after the first week of age. Water and feed were provided ad libitum, and the illumination was 24 h throughout experiment. The initial heating was done by infrared lamps of 250 W, trying to keep the temperature between 28 and 30 °C during the first 2 weeks of life. The broilers were vaccinated against Marek’s disease, Gumboro and fowl pox in the hatchery, followed by vaccination at 5 and 21 days old against Gumboro disease and at 8 days old against Newcastle disease; both vaccines were administered through drinking water. The room temperature and air humidity were controlled, using the handling of curtains and fans to ensure thermal comfort of the broilers.

### Composition of diets and experimental design

The experimental diets were formulated based on corn and soybean meal according Tables for Broilers (Rostagno 2005; Tables 1, 2). The feeding program was divided into two stages: initial (1–21 days old) and growth (22–42 days old). The experimental design was completely randomized in a 2 × 2 + 1 factorial design; two levels of phytase (250 and 500 FTU/kg of feed), two levels of phytic phosphorus (33 and 66 % more than recommended by Tables for Broilers) and a control diet without enzyme and with available phosphorus according Tables for Broilers. The experiment was carried out with 7 replications of 30 broilers and a total of 210 broilers per treatment (Table 3). The results analyzed at 21 and 42 days-old were live weight (LW), weight gain (WG), feed intake (FI), feed conversion (FC), viability (VB) and productive efficiency index (PEI). The VB and PEI were determined according to the following equation:

1 $${\text{VB}}\;(\% ) = \left[ {\frac{{{\text{number}}\;{\text{of}}\;{\text{total}}\;{\text{birds}} - ({\text{number}}\;{\text{of}}\;{\text{dead}}\;{\text{birds}} + {\text{discards}})}}{{{\text{number}}\;{\text{of}}\;{\text{total}}\;{\text{birds}}}} } \right] \times 100$$

2 $${\text{PEI}} = \frac{{{\text{daily}}\;WG\;({\text{g}}) \times VB\;(\% )}}{FC \times 10}$$

**Table 1:** Composition of the experimental diets for broilers from 1 to 21 days old

Ingredients (%)	Treatment^a^				
	T1 (control)	T2	T3	T4	T5
Corn	567.9	567.9	568.9	567.9	569.1
Soybean meal	365.0	365.0	365.0	365.0	365.0
Soybean oil	26.0	26.0	26.0	26.0	26.0
Dicalcium phosphate	17.5	15.5	15.5	13.3	13.3
Limestone	9.0	10.0	10.0	12.0	12.0
NaCl	5.0	5.0	5.0	5.0	5.0
Premix^®^	2.0	2.0	2.0	2.0	2.0
l-Methionine	3.0	3.0	3.0	3.0	3.0
l-Lysine	2.5	2.5	2.5	2.5	2.5
l-Threonine	0.8	0.8	0.8	0.8	0.8
Choline chloride	0.8	0.8	0.8	0.8	0.8
Coccidiostat	0.5	0.5	0.5	0.5	0.5
Inert	0.0	1.0	0.0	1.2	0.0

	Calculated values				
Metabolizable energy (kcal/kg)	3.000	3.000	3.000	3.000	3.000
Crude protein	214.6	214.6	214.6	214.6	214.6
Calcium	9.130	9.130	9.130	9.130	9.130
Choline chloride	0.560	0.560	0.560	0.560	0.560
Available phosphorus	4.371	4.371	4.371	4.371	4.371
Total phosphorus	7.000	6.615	6.615	6.615	6.615
Sodium	2.200	2.200	2.200	2.200	2.200
Digestible lysine	12.400	12.400	12.400	12.400	12.400
Digestible methionine	6.038	6.038	6.038	6.038	6.038
Digestible methionin + cystine	8.900	8.900	8.900	8.900	8.900
Digestible threonine	8.110	8.110	8.110	8.110	8.110
Digestible tryptophan	2.489	2.489	2.489	2.489	2.489
Digestible valine	8.887	8.887	8.887	8.887	8.887
Digestible arginine	13.521	13.521	13.521	13.521	13.521
Digestible isoleucine	8.467	8.467	8.467	8.467	8.467

Enrichment per kilogram of diet: retinol, 25 mg; cholecalciferol, 0.5 mg; dl-tocopheryl acetate, 20 mg; phylloquinone, 0.5 mg; thiamin, 2 mg; riboflavin, 3.6 mg; cyanocobalamin, 20 µg; calcium pantothenate, 10 mg; folic acid, 0.5 mg; growth promoter, 50 mg; niacin, 100 mg; Cu, 75 mg; I, 1.25 mg; Se, 0.25 mg; Mn, 120 mg; Zn, 100 mg; Fe, 50 mg; antioxidant, 0.5 mg; coccidiostat, 110 mg

^a^T1—control diet without enzyme and with available phosphorus according Tables for Broilers (Rostagno 2005); T2—250 FTU/kg of feed and 33 % more phytic phosphorus than T1; T3—500 FTU/kg of feed and 33 % more phytic phosphorus than T1; T4—250 FTU/kg of feed and 66 % more phytic phosphorus than T1; T5—500 FTU/kg of feed and 66 % more phytic phosphorus than T1; FTU—phytasic unit

**Table 2:** Composition of the experimental diets for broilers from 22 to 42 days old

Ingredients (%)	Treatment^a^				
	T1 (control)	T2	T3	T4	T5
Corn	660.4	661.5	6615	662.2	662.2
Soybean meal	303.0	303.0	303.0	303.0	303.0
Soybean oil	0.0	0.0	0.0	0.0	0.0
Dicalcium phosphate	15.8	13.7	13.7	11.7	11.7
Limestone	9.0	10.0	10.0	11.3	11.3
NaCl	5.0	5.0	5.0	5.0	5.0
Premix^®^	2.0	2.0	2.0	2.0	2.0
l-Methionine	2.2	2.2	2.2	2.2	2.2
l-Lysine	2.1	2.1	2.1	2.1	2.1
l-Threonine	0.0	0.0	0.0	0.0	0.0
Choline chloride	0.0	0.0	0.0	0.0	0.0
Coccidiostat	0.5	0.5	0.5	0.5	0.5
Inert	0.0	0.0	0.0	0.0	0.0

	Calculated values				
Metabolizable energy (kcal/kg)	2.950	2.953	2.953	2.955	2.955
Crude protein	195.0	195.0	195.0	195.0	195.0
Calcium	8.300	8.300	8.300	8.300	8.300
Choline chloride	0.00	0.00	0.00	0.00	0.00
Available phosphorus	3.967	3.967	3.967	3.967	3.967
Total phosphorus	6.500	6.132	6.132	5.764	5.764
Sodium	2.200	2.200	2.200	2.200	2.200
Digestible lysine	10.700	10.700	10.700	10.700	10.700
Digestible methionine	4.992	4.992	4.992	4.991	4.991
Digestible methionin + cystine	7.700	7.700	7.700	7.700	7.700
Digestible threonine	6.574	6.574	6.574	6.574	6.574
Digestible tryptophan	2.187	2.186	2.186	2.186	2.186
Digestible valine	8.058	8.058	8.058	8.057	8.057
Digestible arginine	11.943	19.41	19.41	11.939	11.939
Digestible isoleucine	7.544	7.543	7.543	7.542	7.542

Enrichment per kilogram of diet: retinol, 25 mg; cholecalciferol, 0.38 mg; dl-tocopheryl acetate, 20 mg; phylloquinone, 0.5 mg; thiamin, 2 mg; riboflavin, 3.6 mg; cyanocobalamin, 20 µg; calcium pantothenate, 8 mg; folic acid, 0.3 mg; growth promoter, 50 mg; niacin, 30 mg; Cu, 40 mg; I, 1.25 mg; Se, 0.25 mg; Mn, 80 mg; Zn, 80 mg; Fe, 35 mg; antioxidant, 0.5 mg; coccidiostat, 110 mg

^a^T1—control diet without enzyme and with available phosphorus according Tables for Broilers (Rostagno 2005); T2—250 FTU/kg of feed and 33 % more phytic phosphorus than T1; T3—500 FTU/kg of feed and 33 % more phytic phosphorus than T1; T4—250 FTU/kg of feed and 66 % more phytic phosphorus than T1; T5—500 FTU/kg of feed and 66 % more phytic phosphorus than T1; FTU—phytasic unit

**Table 3:** Content of phosphorus from corn and soybean meal for feed formulation

	Tables for broilers^a^	33 % phosphorus	66 % phosphorus
Corn (%)			
Available phosphorus	0.08	0.11	0.13
Total phosphorus	0.24	0.24	0.24
Soybean meal (%)			
Available phosphorus	0.21	0.28	0.35
Total phosphorus	0.65	0.65	0.65

^a^Rostagno (2005)

### Determination of carcass yield and parts

At the end of the experiment, at 42 days after a fasting period of 6 h, 2 broilers were slaughtered per experimental unit were selected (14 per treatment). The broilers were weighed before slaughtered to obtain the body weight. After weighing the carcass, they were cut to evaluate carcass yield (excluding head, neck and feet), breast yield, yield leg (thigh + drumstick), wing back yield and yield.

### Evaluation of bones

At the end of the experiment, 1 bird per experimental unit was selected (7 per treatment) and was sacrificed to extract tibia and femur. These bones were used for the analysis of dry matter, mineral matter, calcium and phosphorus. First, the bones were cleaned to remove soft tissue and treated with ethyl ether by 8 h. Then, the samples were dried at 105 °C by 12 h, weighed and placed in a muffle furnace at 600 °C for 4 h to yield ash. Then the samples were prepared for analysis of minerals (Sakomura and Rostagno 2007).

### Reproducibility and statistical analyses of results

The statistical analyses were performed using the SISVAR Software 5.1 Build 72 (Ferreira 2011) and/or GraphPad Prism 6 Software. In the case of statistical significance, the means were compared according to the Student Newman Kwels (SNK) test.

## Results and discussion

### Analyses of mycotoxin and cytotoxicity of crude phytase from *A. japonicus*

**Fig. 1:**
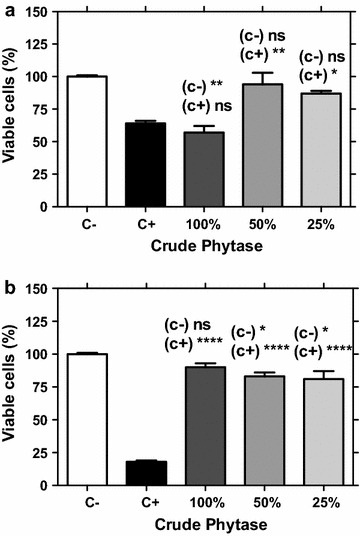
Analysis of cytotoxicity (**a**) and cellular surviving (**b**) of cells treated with crude phytase from *A. japonicus*. *Symbols*: *C−* negative control—saline 0.9 %, *C+* positive control—doxorubicin 0.3 µg/mL, *ns* not significant, *stars* level of significance (*p* < 0.05)

The cellular viability of MRC-5 cell line was assessed after 24 and 120 h of exposure to high concentrations of the crude phytase (100, 50, and 25 % [v/v]). Figure 1a shows that treatments with 50 and 25 % (v/v) of sample resulted in 93 and 87 % of viable cells, respectively, after 24 h of treatment. These data indicated low toxicity of these concentrations. The ANOVA analysis showed no statistically significant difference between negative control and treatments with 50 and 25 % (v/v) of sample (*p* > 0.05), but the treatment with 100 % (v/v) showed significant difference compared to negative control (*p* < 0.05). In fact, the assay treated with 100 % (v/v) of crude phytase showed cytotoxicity next to that observed in the positive control (*p* > 0.05).

The cellular viability measured after 120 h showed that all treatments had more than 80 % of viable cells (Fig. 1b). According to the ANOVA analysis, the treatment with 100 % (v/v) of crude phytase showed no statistically significant difference (*p* > 0.05) relative to negative control, although the treatments with 50 and 25 % (v/v) showed a few significant differences (*p* < 0.05). All treatments had high statistically significant difference (*p* < 0.05) relative to positive control. These results confirmed the safe concentration of 50 % (v/v). The concentration added on animal diet was posteriorly estimated to be ranging from 2 to 4 % (v/w) (20–40 mL/kg of animal food), and certainly would not lead to toxic effects in animals.

In order to confirm the safety of crude phytase, the extract was sent to JLA Brasil Laboratory to quantify mycotoxins. The report n# C00009398, delivered by this company, revealed no traces of aflatoxin B1, B2, G1 and G2 and zearalenone and ochratoxin A in the sample. Thus, the crude phytase would not have any toxic effect when applied to chicken feed; it may liberate phosphate from phytate and improve animal development.

### Effect on performance of broiler feed treated with phytase

The results analyzed were feed intake, weight gain and feed conversion for 21-days-old and 42-days-old animals. The performance results showed no statistically significant difference between the control treatment and any treatment with phytase (Table 4). The treatments with or without phytase, regardless of the enzyme concentration (250–500 FTU), and normal or reduced levels of inorganic phosphate in the diet showed no difference of results analyzed.

**Table 4:** Performance of 21-days-old and 42-days-old broilers

Treatment^a^	Performance					
	Feed intake (kg)		Weight gain (kg)		Feed conversion	
	21-days-old	42-days-old	21-days-old	42-days-old	21-days-old	42-days-old
T1 (control)	1.27a	4.38a	0.87a	2.62a	1.45a	1.68a
T2	1.27a	4.44a	0.87a	2.61a	1.46a	1.70a
T3	1.28a	4.44a	0.86a	2.64a	1.49a	1.68a
T4	1.25a	4.45a	0.85a	2.63a	1.46a	1.69a
T5	1.22a	4.38a	0.85a	2.59a	1.44a	1.69a
ANOVA	ns^b^	ns	ns	ns	ns	ns
CV^c^ (%)	4.48	3.91	3.29	3.29	3.80	2.96

Different lowercase letters in columns show significant level (*p* < 0.05) according Student Newman Kwels test (SNK)

^a^T1—control diet without enzyme and with available phosphorus according Tables for Broilers (Rostagno 2005); T2—250 FTU/kg of feed and 33 % more phytic phosphorus than T1; T3—500 FTU/kg of feed and 33 % more phytic phosphorus than T1; T4—250 FTU/kg of feed and 66 % more phytic phosphorus than T1; T5—500 FTU/kg of feed and 66 % more phytic phosphorus than T1; FTU—phytasic unit

^b^ns—not significant with 5 % of significance

^c^CV—coefficient of variance

The live weight, viability and productive efficiency index were analyzed for 42-days-old animals. The analyses did not show any statistically significant difference either (Table 5). The similar effect can be observed in carcass yield (Table 6). All parts analyzed in this experiment did not show statistically significant difference of yield between treatment assays and control assay.

**Table 5:** Final performance of 42-days-old broilers

Treatment^a^	Performance		
	Live weight (kg)	Viability (kg)	Productive efficiency index
T1 (control)	2.663a	99.05a	377a
T2	2.609a	97.62a	361a
T3	2.604a	96.67a	371a
T4	2.644a	98.10a	370a
T5	2.567a	97.62a	357a
ANOVA	ns^b^	ns	ns
CV^c^ (%)	4.83	2.33	5.38

Different lowercase letters in columns show significant level (*p* < 0.05) according Student Newman Kwels test (SNK)

^a^T1—control diet without enzyme and with available phosphorus according Tables for Broilers (Rostagno 2005); T2—250 FTU/kg of feed and 33 % more phytic phosphorus than T1; T3—500 FTU/kg of feed and 33 % more phytic phosphorus than T1; T4—250 FTU/kg of feed and 66 % more phytic phosphorus than T1; T5—500 FTU/kg of feed and 66 % more phytic phosphorus than T1; FTU—phytasic unit

^b^ns—not significant with 5 % of significance

^c^CV—coefficient of variance

**Table 6:** Body measurements of 42-days-old broilers

Treatment^a^	Carcass yield^d^ (g/kg)				
	Carcass yield	Thigh + drumstick yield	Breast yield	Back yield	Wing yield
T1 (control)	744.0a	288.9a	400.0a	206.3a	103.9a
T2	738.1a	288.7a	397.4a	207.5a	103.0a
T3	748.5a	282.8a	390.4a	215.4a	101.9a
T4	757.4a	278.1a	384.0a	212.2a	103.6a
T5	728.9a	287.0a	404.0a	207.1a	113.4a
ANOVA	ns^b^	ns	ns	ns	ns
CV^c^ (%)	3.87	6.06	5.45	7.14	11.14

Different lowercase letters in columns show significant level (*p* < 0.05) according Student Newman Kwels test (SNK)

^a^T1—control diet without enzyme and with available phosphorus according Tables for Broilers (Rostagno 2005); T2—250 FTU/kg of feed and 33 % more phytic phosphorus than T1; T3—500 FTU/kg of feed and 33 % more phytic phosphorus than T1; T4—250 FTU/kg of feed and 66 % more phytic phosphorus than T1; T5—500 FTU/kg of feed and 66 % more phytic phosphorus than T1; FTU—phytasic unit

^b^ns—not significant with 5 % of significance

^c^CV—coefficient of variance

^d^Eviscerated carcass without feet, head and neck

These results support the work described in the literature (Lelis et al. 2010). The authors evaluated the addition of two levels of phytase in diets based on corn and soybean meal. The diets were formulated to provide a nutritional matrix recommended for broilers from 1 to 40 days, with the addition of enzyme. As in our study, animals fed with dietary treatments supplemented with phytase maintained the same performance of broilers fed according to the dietary recommendations. Therefore, phytase supplementation could supply the deficiency of protein and available phosphorus in diets tested.

Also, the reduction of non-phytic phosphate to 70 % of the amount required by the animals caused a decrease in weight gain, feed intake and feed conversion, and the addition of phytase increased weight gain and feed intake in 21-days-old animals (Oliveira et al. 2009) In the same way, the phytase improved the development of broilers from 8 to 22 days (Persia and Saylor 2006). However, the literature also describes that there was no improvement in the performance of broiler feed diets supplemented with two levels of commercial phytase in initial (1–21 days) and growth (22–42 days) phases (Lima et al. 2002). Besides, the phytase did not affect broiler production with or without addition of rice polishing (Salinas-Chavira et al. 2012).

Our results indicate that the action of phytase from *A. japonicus* promoted a positive effect on inorganic phosphorus-depleted feed, resulting in an animal development similar to animals treated with commercial feed. In addition to the phytase effect, this improvement may be related to the activity of other enzymes secreted by the *A. japonicus*, such as glycoside hydrolases (Yin et al. 2007; Mussatto et al. 2009; Wakiyama et al. 2010).

### Effect of the food treatments on bones of broilers

The dry matter, mineral matter, and calcium and phosphorus of femur and tibia of animals at 42 days of age were measured to verify the specific effect of phytase on feed supplementation.

The analysis of femur showed significant effect in the measurement of phosphorus from dry matter and total mineral matter (*p* < 0.05; Table 7). The highest percentage of phosphorus from dry matter is observed in T1 (control), T2 (250 FTU/kg plus 33 % of inorganic phosphate) and T4 (250 FTU/kg plus 66 % of inorganic phosphate). The same result can be observed for total mineral matter. For quantification of total dry matter, calcium and phosphorus from mineral matter, there was no statistically significant difference between treatments (*p* > 0.05).

**Table 7:** Biochemical analysis of calcium and phosphorus of femur of the 42-days-old broilers

Treatment^a^	Femur					
	Dry matter (g/kg)			Mineral matter (g/kg)		
	Total	Calcium	Phosphorus	Total	Calcium	Phosphorus
T1 (control)	590.9a	191.8a	95.7a	538.3a	356.4a	177.8a
T2	584.2a	187.8a	93.4a	540.5a	347.7a	173.0a
T3	557.9a	184.0a	88.4b	499.8b	368.3a	176.7a
T4	573.2a	179.7a	93.1a	528.3a	340.1a	176.3a
T5	564.1a	178.0a	86.4b	514.2ab	348.9a	168.7a
ANOVA	ns^b^	ns	p < 0.05	p < 0.05	ns	ns
CV^c^ (%)	4.36	5.09	3.77	3.82	6.63	3.92

Different lowercase letters in columns show significant level (*p* < 0.05) according Student Newman Kwels test (SNK)

^a^T1—control diet without enzyme and with available phosphorus according Tables for Broilers (Rostagno 2005); T2—250 FTU/kg of feed and 33 % more phytic phosphorus than T1; T3—500 FTU/kg of feed and 33 % more phytic phosphorus than T1; T4—250 FTU/kg of feed and 66 % more phytic phosphorus than T1; T5—500 FTU/kg of feed and 66 % more phytic phosphorus than T1; FTU—phytasic unit

^b^ns—not significant with 5 % of significance

^c^CV—coefficient of variance

The analysis of the tibia showed significant effect on the quantification of total dry matter, total mineral matter and calcium from mineral matter (*p* < 0.05; Table 8). The T1 (control), T2 (250 FTU/kg plus 33 % of inorganic phosphate), T3 (500 FTU/kg plus 33 % of inorganic phosphate) and T4 (250 FTU/kg plus 66 % of inorganic phosphate) showed the highest amounts of total dry matter. Besides, T1 (control) and T2 (250 FTU/kg plus 33 % of inorganic phosphate) showed the highest values of total mineral matter. However, T5 (500 FTU/kg plus 66 % of inorganic phosphate) showed the highest amount of calcium from mineral matter. Other variables, such as calcium and phosphorus from dry matter and phosphorus from mineral matter, showed no significant effect between treatments (*p* > 0.05).

**Table 8:** Biochemical analysis of calcium and phosphorus of tibia of 42-days-old broilers

Treatment^a^	Tibia					
	Dry matter (g/kg)			Mineral matter (g/kg)		
	Total	Calcium	Phosphorus	Total	Calcium	Phosphorus
T1 (control)	613.9a	192.6a	95.8a	538.3a	357.9b	178.0a
T2	616.3a	189.9a	95.1a	539.0a	352.5b	176.4a
T3	610.1a	187.7a	93.5a	526.0ab	356.9b	177.8a
T4	613.6a	177.1a	93.3a	526.4ab	338.4b	178.2a
T5	591.8b	195.8a	94.1a	505.3b	387.1a	185.7a
ANOVA	p < 0.05	ns^b^	ns	p < 0.05	p < 0.05	ns
CV^c^ (%)	3.99	6.63	6.03	3.27	5.45	3.85

Different lowercase letters in columns show significant level (*p* < 0.05) according Student Newman Kwels test (SNK)

^a^T1—control diet without enzyme and with available phosphorus according Tables for Broilers (Rostagno 2005); T2—250 FTU/kg of feed and 33 % more phytic phosphorus than T1; T3—500 FTU/kg of feed and 33 % more phytic phosphorus than T1; T4—250 FTU/kg of feed and 66 % more phytic phosphorus than T1; T5—500 FTU/kg of feed and 66 % more phytic phosphorus than T1; FTU—phytasic unit

^b^ns—not significant with 5 % of significance

^c^CV—coefficient of variance

According to the analyses of bones, crude phytase could supply the need for phosphate in diets deficient in this mineral. Furthermore, the enzyme favored the formation of bone structure and performance of the broilers, since a good bone support results in an increase in muscle development. Thus, mineralization and bone strength were parameters that showed the efficacy of phytase of *A. japonicus* to provide phosphate and other minerals for broilers from the feed. The literature describes the evaluation of four levels of available phosphorus from corn and soybean meal (34, 56, 78 and 100 % availability) and three levels of phytase (0, 500 and 1000 FTU/kg feed; Laurentiz et al. 2007). The authors observed that the levels of available phosphate affected the contents of ash, phosphorus, manganese and zinc in the tibias of broilers. The addition of phytase resulted in a significant increase in the contents of ash, phosphorus and zinc. Besides, Oliveira et al. (2009) analyzed two levels of phytase (0 and 25 FTU/kg) and three levels of available phosphorus (70, 85 and 100 % of requirements). The authors observed no negative effect (*p* > 0.05) of lower levels of available phosphate on phosphorus and mineral content of the tibia when phytase was added to the feed.

## Conclusions

Then, this work studied the safe supplementation of phytase from *A. japonicus* in young chicken feed and verified the biochemical effect on performance and bones of these birds. The enzyme was thermostable up to 50 °C, presented a t_50_ of 40 min, and it has more than 80 % of activity at 41–42 °C and acid pH, conditions found in digestive tract, which indicated a good performance of this enzyme in supplementation of birds feed. Low concentrations of crude phytase from *A. japonicus* do not have cytotoxicity effects on cells derived from lung tissue and have no mycotoxins. Furthermore, the supplementation of broilers diet increases the inorganic phosphate releasing and overcomed the deficiency of this compound in the food formulation. Thus, dietary supplementation with crude phytase reached zootechnical indexes required for the development of the animals. So, *A. japonicus* phytase could be used as a new commercial alternative to animal diet supplementation.
